# A New Method of Identifying Core Designers and Teams Based on the Importance and Similarity of Networks

**DOI:** 10.1155/2021/3717733

**Published:** 2021-07-20

**Authors:** Dianting Liu, Kangzheng Huang, Danling Wu, Shenglan Zhang

**Affiliations:** ^1^College of Mechanical and Control Engineering, Guilin University of Technology, Guilin 541004, China; ^2^College of Information Science and Engineering, Guilin University of Technology, Guilin 541004, China

## Abstract

In the process of product collaborative design, the association between designers can be described by a complex network. Exploring the importance of the nodes and the rules of information dissemination in such networks is of great significance for distinguishing its core designers and potential designer teams, as well as for accurate recommendations of collaborative design tasks. Based on the neighborhood similarity model, combined with the idea of network information propagation, and with the help of the ReLU function, this paper proposes a new method for judging the importance of nodes—LLSR. This method not only reflects the local connection characteristics of nodes but also considers the trust degree of network propagation, and the neighbor nodes' information is used to modify the node value. Next, in order to explore potential teams, an LA-LPA algorithm based on node importance and node similarity was proposed. Before the iterative update, all nodes were randomly sorted to get an update sequence which was replaced by the node importance sequence. When there are multiple largest neighbor labels in the propagation process, the label with the highest similarity is selected for update. The experimental results in the related networks show that the LLSR algorithm can better identify the core nodes in the network, and the LA-LPA algorithm has greatly improved the stability of the original LPA algorithm and has stably mined potential teams in the network.

## 1. Introduction

The design tasks of some enterprises only allow the participation of designers within the enterprise and do not support the participation of many designers on the network [1]. However, this situation makes it impossible for companies to make full use of the experience accumulated by professionals from different fields and with different experiences on the Internet and cannot fully learn from the wisdom of the outside of the company. With the development of the Internet, the substantial growth of network users has given birth to the huge potential of the Internet. And crowdsourcing, an emerging collaborative approach, is emerging. More and more companies outsource the tasks previously completed by their internal designers to nonspecific virtual communities in a free and voluntary form [2]. The task of crowdsourcing is generally applied by the designer, but for some difficult tasks, the designer needs to cooperate to complete the realization.

The designer network is a network that takes the designer as the node and the relationship between the designers as the edge. This network not only reflects the relationship between designers but also reflects the social relationship between people in society to a certain extent. The association between designers is abstracted as a network. The small-world characteristics of the network [3] and the scale-free characteristics [4] make some special nodes in the network have a great influence on the performance and structure of the network. These nodes are called important nodes, and they are the core designers in the designer network. Therefore, how to quickly evaluate the importance of nodes in the network and mine the key nodes is of great significance [5].

Researchers have done a lot of work in this area and proposed many classic node importance evaluation algorithms [6], including Degree Centrality, Betweenness Centrality, Closeness Centrality, Eigenvector Centrality, and the K-shell decomposition algorithm [7–11]. In recent years, algorithms combining multiple attributes have emerged to measure the importance of nodes in complex networks. The local centrality (LC) algorithm proposed by Chen et al. [12] comprehensively considers the importance of the degree information of the node itself and its neighbors as a node in the network; Wang et al. [13] believed that the importance of a node was related to its own degree and the degree of neighboring nodes; that is, the greater the degree of neighboring nodes of a node, the more important the node (WL); Lü et al. [14] introduced the H-index [15], which is used to evaluate the influence of academic paper authors, into the field of node importance analysis in complex networks; Ruan et al. [5] quantified the local network topology coincidence degree of nodes and considered the node degree and neighbor node topology coincidence degree to characterize its structural importance in the network (LLS).

It is a hot topic to study how to improve the robustness of complex networks and systems. On the one hand, the performance of the entire network can be enhanced by protecting key nodes; on the other hand, new coupling strategies can be proposed to improve the robustness of the network. For example, Wang et al. [16] considered the propagation process of cascading failures when calculating failed nodes and proposed a new coupling strategy to improve the robustness of the network. Reference [17] found that the coupled network is more robust than a single artificial network under random attacks.

In the real world, the identification of important nodes is not only related to the network structure but also related to factors such as trust in network propagation. People are often more willing to listen to the opinions of people they trust; that is, trusting neighbors will more or less affect people's decision-making. Based on this, the idea can be applied to further distinguish the importance of different nodes. Based on the existing algorithms for calculating the importance of nodes, by using the activation function ReLU [18], a local centrality method based on the ReLU function, LLSR, is proposed to measure the importance of nodes in the network.

The designer's cooperative behavior is generated and maintained through the formation of close collaborator clusters through network reciprocity [19, 20]. A potential designer team is a group of designers who cooperate closely and can be understood as a community in the network. Members in the same community cooperate closely, and members in different communities are sparsely connected. The community is a group, and this characteristic accords with the high aggregation and sociality of human behavior. Aiming at the problem of how to find potential designer teams for difficult tasks, community detection algorithms are used to find potential designer teams.

The community is the basic structure of the network. Exploring the potential community and its structure in the network is of great significance for the study of the rule of information dissemination and the accurate recommendation of collaborative design tasks. In recent years, community detection algorithms have been widely proposed, which has aroused widespread interest among researchers [21]. LPA mainly uses the propagation characteristics of the network to affect the label of each node, so as to detect the community structure in the network; it has an approximately linear time complexity and has achieved good results in many networks. Due to the random selection process in LPA, it is mainly manifested in the order of node label update and the large randomness in the label propagation process, which may lead to a large community, which does not accord with the actual situation. Therefore, many scholars have proposed improved algorithms. Zheng and Yue [22] aimed for the instability of the algorithm; an improved algorithm using random walk was proposed. The neighbor influence is calculated and the label is updated in ascending order [23]. Gui et al. [24] aimed for the problem of update randomness in label propagation; an improved algorithm using boundary nodes was proposed. In order to solve the random selection process in LPA, a new label propagation algorithm based on node importance and node similarity (LA-LPA) is proposed, and the node importance sequence (LLSR) is used to replace the randomized node sequence. In order to solve the randomness in the propagation process, the node label with the highest Adamic-Adar similarity among neighbor nodes is selected.

Our main contributions are summarized as follows:

1. We propose a local centrality algorithm LLSR based on the ReLU function to measure the importance of nodes in the network
2. Aiming at the problem of updating node labels by randomizing the order of nodes in the LPA algorithm and the problem of randomly selecting labels when multiple maximum neighbor labels appear during updating labels, we propose LA-LPA algorithm based on node importance LLSR and node similarity Adamic-Adar, and node labels are updated with the node importance ranking obtained by LLSR algorithm; when multiple maximum neighbor labels appear during updating the label, the Adamic-Adar coefficient is introduced into the LPA algorithm, and the node label with the highest Adamic-Adar similarity is selected
3. We performed experiments on the data sets to prove the effectiveness of the LLSR algorithm and the LA-LPA algorithm

The rest of this paper is organized as follows. In Section 2, we introduce the theoretical basis of node importance, the LPA algorithm, and its defects. In Section 3, we introduce the process of improving the node importance algorithm and improving the LPA algorithm, propose the node importance algorithm LLSR and the node similarity index based on local information, and propose the improved LA-LPA algorithm based on the node importance algorithm and node similarity. In Section 4, we introduce the evaluation criteria of the important nodes and community detection. In Section 5, we test and compare the Degree, WL, H, LC, LLS, and the improved algorithm LLSR in the real network and the artificial network and test and compare LPA, S-LPA, and the improved algorithm LA-LPA in the real network. Finally, in Section 6, we present our conclusions and discuss the results.

## 2. Related Theories

Suppose that the network *G* (*V*, *E*) is an undirected and unweighted complex network, where *V*={*v*_1_, *v*_2_,…, *v*_*n*_} represents the node set, with |*V*|=*n* nodes; *E*={*e*_1_, *e*_2_,…, *e*_*n*_} represents edge set, with |*E*|=*m* edges; A represents the adjacency matrix of graph *G*; when nodes *i* and *j* are connected, *A*_*ij*_=1; otherwise, *A*_*ij*_=0.

### 2.1. Theoretical Basis of Node Importance

(1) *Degree Centrality.* Degree centrality refers to the number of connected edges of a node *v*_*i*_, that is, the number of neighbor nodes of the node *v*_*i*_, and its calculation equation is as follows:
  (1) $$\begin{array}{c} \text{DC}\left(v_{i}\right)=\sum_{j=1}^{N}A_{ij}. \end{array}$$
(2) *Betweenness Centrality*. Betweenness centrality is the ratio of the number of shortest paths between any two nodes through the node to the number of all shortest paths:
  (2) $$\begin{array}{c} \text{BC}\left(v_{i}\right)=\sum_{i\neq j\neq k}\frac{\sigma \left(j,k|i\right)}{\sigma \left(j,k\right)}, \end{array}$$
where  *σ*(*j*, *k|i*) represents the number of shortest paths from node *j* to node *k* through node *i*, and *σ*(*j*, *k*) is the number of shortest paths from node *j* to node *k*.
(3) *Closeness Centrality*. The closeness centrality is to judge whether the node is in the center of the network by measuring the reciprocal of the sum of the distance between the node and other nodes:
  (3) $$\begin{array}{c} \text{CC}\left(v_{i}\right)=\frac{1}{\sum_{i\in V}d_{ij}}, \end{array}$$
where *d*_*ij*_ is the shortest distance from node *i* to node *j*.
(4) *H-Index Centrality*. H-index centrality determines the importance of a node by considering the degree of the node itself and the degree of neighboring nodes:
  (4) $$\begin{array}{c} H-\text{index}\left(v_{i}\right)=H\left(d_{u_{1}},d_{u_{2}},\ldots ,d_{u_{j}}\right), \end{array}$$
where (*d*_*u*_1__, *d*_*u*_2__,…, *d*_*u*_*j*__) represents the neighbor nodes of node *v*_*i*_, *d*_*u*_*j*__ represents the degree of node *u*_*j*_, and the function *H*(*d*_*u*_1__, *d*_*u*_2__,…, *d*_*u*_*j*__) returns a maximum value *h*, making at least *h* node in *d*_*u*_1__, *d*_*u*_2__,…, *d*_*u*_*j*__ have a degree value greater than or equal to *h*.

The difference in node importance algorithms is mainly reflected in the definition of node importance, and the importance of nodes is determined by the topology of the network [25–27]. Starting from the network topology is one of the common methods to study this problem. To measure the importance of nodes in the network, it is divided into global attributes and local attributes. From a global attribute, the importance of nodes in the network needs to traverse the entire network, which has certain application value in small-scale networks, and the time complexity is too high on large-scale networks. The measurement method based on local information only needs the local topology to measure the importance of nodes. The propagation ability of nodes in the network is related to the importance of nodes. The more important the node, the stronger the propagation ability. A key node is selected as the source of propagation, which allows information to be disseminated more quickly; the product release range is wider, and so on.

### 2.2. Label Propagation Algorithm

LPA mainly uses the propagation characteristics of the network to affect the label of each node, so as to detect the community structure in the network, but the algorithm has instability and randomness.

#### 2.2.1. Concept of Label Propagation Algorithm

The LPA algorithm is as follows:

1. *Initialization.* All nodes in the network are assigned a label, and the label of the node represents the community in which it is located
2. *Label Propagation*. The order of nodes is randomized and updated according to the new order. At time *t*, node *v*_*i*_ receives the label by all its neighbor nodes at time *t* − 1 and then updates its own label in its neighbor nodes with the maximum frequency; if more than one label with the highest frequency appears, one of these labels with the highest frequency will be randomly selected
3. *Convergence Condition*. When the labels of all nodes remain unchanged or the set number of iterations is reached, the algorithm stops
4. *Community Detection*. Count the labels of each node, and nodes with the same label content are classified as the same community, and the number of label types indicates the number of communities

#### 2.2.2. Disadvantages of Label Propagation Algorithm

The LPA algorithm has linear time complexity and does not need to set the number of communities in advance and is suitable for community detection in large-scale networks. However, LPA assigns a unique label to each node before the start of the iteration and obtains a random node update sequence. When there is more than one label with the highest frequency in the neighbor nodes, a label will be randomly selected from the labels with the highest frequency. Due to the random selection process in LPA, this process affects the accuracy and stability of the LPA algorithm for community detection to a certain extent, and community annexation may occur.

**Figure 1:**
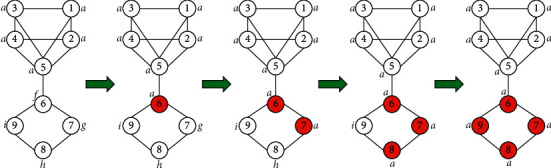
Community annexation.

When the labels of all nodes in a large community stop spreading, if the labels of neighboring small communities have not spread, the label of the large community will affect the label of the small community, resulting in the annexation of the small community, which may eventually form a giant community, which makes the quality of the community detection results very poor. As shown in Figure 1, it can be seen from the figure that there are two communities in the network. After iterative propagation, the nodes in the upper part belong to the same community. When the nodes in the lower part are updated, node 6 randomly selects one of the labels *a*, *g*, and *i*. If the label of node 5 is selected by node 6, then the labels of all 4 nodes in the lower part are *a*. This led to the annexation of the following communities, and the entire network eventually became a community.

## 3. Improve Algorithm

### 3.1. LLSR Algorithm

#### 3.1.1. LLSR Algorithm Description

The importance of the node in the network depends not only on the degree of the node itself but also on the degree of dependence of neighbor nodes within two hops on the node. By calculating the overlap degree of neighboring nodes in terms of topological structure, the similarity of the node domain is defined. The node similarity index is defined as the Jaccard index value [28], and the calculation of node similarity is shown in

(5) $$\begin{array}{c} \text{sim}\left(b,c\right)=\left\{\begin{array}{cc} \frac{\left|N\left(b\right){}^{\cap }N\left(c\right)\right|}{\left|N\left(b\right){}^{\cup }N\left(c\right)\right|}, & b\text{ and }c\text{ have no edge},\\ 1, & b\text{ and }c\text{ have edge}.\; \end{array}\right. \end{array}$$

The value of sim is between 0 and 1. The smaller the similarity of neighbor nodes is, the more important the node is. If the number of neighbor nodes is large and the degree of overlap of the network topology between them is lower, it indicates that the greater the degree of dependence of the node in the network and the stronger its functional irreplaceability, the greater the importance of the node. Based on the similarity of neighbor nodes, Ruan et al. [5] proposed an important method based on neighborhood similarity, LLS, and its calculation equation is as follows:

(6) $$\begin{array}{c} \text{LLS}\left(i\right)=\sum_{b,c\in N\left(i\right)}\left(1-\text{sim}\left(b,c\right)\right), \end{array}$$

where *N*(*i*) represents the set of neighbor nodes of node *i*. The LLS method comprehensively considers the similarity between the degree of the node and the neighbor nodes. The larger the LLS value, the greater the degree of the node and the lower the degree of overlap between neighbor nodes.

The above method starts from the topological structure of the network, takes into account the importance of neighboring nodes, and ignores some influencing factors in actual network propagation, such as trust and interactive information. Since people's views in daily life are not the same, everyone's attitude toward things is more or less different. From the perspective of information dissemination, individuals have different levels of acceptance of others' opinions when communicating information. Interindividual communication often occurs between individuals who trust each other, and individuals who do not trust will choose not to communicate with them. Inspired by the above, the importance of different nodes can be further distinguished by communicating with neighbor nodes. This paper uses the ReLU function to improve the LLS method and further revises the node importance level in the network. The calculation equation is as follows:

(7) $$\begin{array}{c} \text{ReLU}\left(x\right)=\left\{\begin{array}{cc} x,\; & \text{if }x>0,\\ 0,\; & \text{otherwise,} \end{array}\right.\\ \text{LLSR}\left(i\right)=\sum_{j\in N\left(i\right)}\text{ReLU}\left(\text{LLS}\left(i\right)-\text{LLS}\left(j\right)\right). \end{array}$$

For each node, the node can be regarded as the output layer and its neighbor node as the input layer. Whether the neighbor node can activate the node depends on the importance of the neighbor node. In a network, when the LLS value of the neighbor node is less than the node, it will be output to the node; otherwise, the neighbor node has no influence on it. The importance of complex network nodes obtained by the ReLU function can better reflect the performance of the actual network.

#### 3.1.2. Time Complexity Analysis

Suppose that the number of nodes in the network *G* (*V*, *E*) is *n*, the number of edges is *m*, and the average degree of the network is <*k*>. In this paper, the time complexity when calculating the value of the LLS method first is *O*(*nk*^2^), and calculating the node similarity is a small number for the LLS algorithm, so it can be ignored. Then, according to the ReLU function to modify the time complexity of the results obtained by the LLS algorithm to *O*(*nk*), the time complexity of the LLSR algorithm in this paper is *O*(*n*(*k*^2^+*k*))) ~ *O*(*n*).

This article compares several classic methods for evaluating the importance of nodes in the network, as shown in Table 1.

### 3.2. LA-LPA Algorithm

#### 3.2.1. Node Similarity Indicator

In the existing node similarity index method, the node similarity focuses on two different perspectives of local information and global information. However, the time complexity based on global information is relatively high, and it has a certain application value in small-scale networks; some node similarity calculation methods based on local information only need local topology information. There is a very important concept in the node similarity method based on local information, that is, common neighbor. The number of common neighbors is calculated by

(8) $$\begin{array}{c} Z_{ij}=\left|\Gamma \left(i\right)\cap \right|\Gamma \left(j\right). \end{array}$$

In equation (8), Γ(*i*) is the set of neighbor nodes of node *i*, and |Γ(*i*)| is the number of neighbor nodes of node *i*. Common neighbors define similarity through the local topology of the network. For example, in a designer network, if two people have more cocollaborators, then their similarity will be higher. They may become a new partner because they are more likely to have met in life. In the Jaccard coefficient, the importance of common neighbor nodes is not considered different. In the designer network, some designers are more important for calculating similarity, while some designers (such as fringe nodes) are of little significance when calculating similarity. The Adamic-Adar coefficient [29] emphasizes the importance of different nodes by considering the degree of the node. The smaller the degree of the node, the greater the weight of the node in the common neighbor. The calculation Equation of the Adamic-Adar coefficient is shown in

(9) $$\begin{array}{c} \text{Adamic}-\text{Adar}\left(x,y\right)=\sum_{z\in \Gamma \left(x\right)\cap \Gamma \left(y\right)}\frac{1}{\log\left|\Gamma \left(z\right)\right|}. \end{array}$$

#### 3.2.2. LA-LPA Algorithm Based on Node Importance LLSR and Node Similarity Adamic-Adar

In LPA, all nodes are randomly sorted before iterative update to get an update sequence, because each time is randomly sorted. This process affects the accuracy and stability of the LPA algorithm for community detection to a certain extent. The LLSR node importance method proposed in this paper obtains the importance of each node and sorts it, obtains the update sequence of the node, and updates the label of the node with this sequence, which can reduce the instability of community detection results in the original LPA due to the randomness of the update sequence. When there are multiple maximum frequency neighbor labels, a label will be randomly selected. In order to solve the randomness in the propagation process, when the above situation occurs, the node label with the highest Adamic-Adar similarity among neighbor nodes is selected. This improvement can effectively prevent the instability of the algorithm and improve the accuracy of community detection. LA-LPA propagates labels according to the rules of

(10) $$\begin{array}{c} v_{i}\left(t\right)=F\left(f\left(v_{i_{1}}\left(t-1\right),v_{i_{2}}\left(t-1\right),\ldots ,v_{i_{k}}\left(t-1\right)\right)\right). \end{array}$$

In equation (10), the function *f* returns the label with the highest frequency among all neighbors of node *v*_*i*_, and function F returns the label of the node corresponding to the label with the highest frequency that is most similar to node *v*_*i*_.

#### 3.2.3. Time Complexity Analysis

From the time complexity of the algorithm, the time complexity of the LLSR algorithm in this paper is *O*(*n*(*k*^2^+*k*)) ~ *O*(*n*), the time required for node label initialization is *O*(*n*); for each node *i* in the network, the time complexity of updating the node label is *O*(*k*), and the time required for one iteration is *O*(*nk*). Therefore, through analysis, the time complexity of the LA-LPA algorithm is about *O*(*n*(*k*^2^+*k*)+*n*+*nk*) ~ *O*(*n*).

## 4. Evaluation Criteria

### 4.1. Node Importance Evaluation Criteria

Monotonicity [30], network-based propagation dynamics model [31], and methods based on network robustness and vulnerability [32] are often used to evaluate the importance of nodes. In different evaluation models, the meaning of node importance is different. Monotonicity is to test the distinguishing ability of different node importance ranking algorithms; in the SIR model of the propagation dynamics model, the importance of a node is determined by the average propagation range of the node. This paper evaluates the node importance method based on the robustness and vulnerability of the network. It mainly studies the largest connected subgraph in the infiltration flow. The coefficient of the largest connected subgraph and the network efficiency index are used to quantify the effect on the network structure and function after the node is removed, so as to evaluate the structural importance of the node.

#### 4.1.1. Largest Connectivity Coefficient

Sort the different node importance ranking methods from large to small, and observe the effect of removing some nodes on the largest connected subgraph of the network. The calculation equation is as follows:

(11) $$\begin{array}{c} G=\frac{R}{N}. \end{array}$$

Among them, *R* represents the number of nodes in the largest connected subgraph remaining in the network after removing some of the nodes. The more obvious the tendency of the largest connected subgraph to decrease with the removal of nodes, the better the effect of attacking the network by this sorting method.

#### 4.1.2. Network Efficiency

Network efficiency is to examine the impact of removing nodes on the network [5]. Removing nodes in the network and all their corresponding edges will increase the average path length of the entire network and affect the connectivity of the network. The network efficiency calculation equation is as follows:

(12) $$\begin{array}{c} \mu =\frac{1}{N\left(N-1\right)}\sum_{i,j\in V}\frac{1}{d_{ij}}. \end{array}$$

This paper simulates the network attack situation by deleting nodes in the network according to different node importance algorithms and calculates the percentage of network efficiency reduction before and after the network is deliberately attacked to measure the accuracy of different node importance algorithms. The network efficiency reduction rate is calculated according to

(13) $$\begin{array}{c} \varepsilon =1-\frac{\mu }{\mu_{0}}. \end{array}$$

*μ* represents the network efficiency after the node is removed, and *μ*_0_ represents the original network efficiency; the larger the value of *ε* is, the worse the network efficiency becomes after the node is removed.

### 4.2. Evaluation Criteria for Community Detection

Modularity (*Q*) [33] is an index proposed by Newman to measure the quality of community detection.

(14) $$\begin{array}{c} Q=\frac{1}{2m}\sum_{ij}\left(A_{ij}-\frac{k_{i}k_{j}}{2m}\right)\delta \left(C_{i},C_{j}\right),\\ m=\frac{1}{2}\sum_{ij}A_{ij}. \end{array}$$

Here, *k*_*i*_ represents the sum of the weights of the edges connecting node *i* and all other nodes. In an undirected graph, when the value of *A*_*ij*_ is 0, it means that there is no edge between node *i* and node *j*; otherwise, it means the connection weight between node *i* and node *j*; *C*_*i*_ represents the community to which node *i* belongs, and *δ*(*C*_*i*_, *C*_*j*_) is a binary function. If *C*_*i*_ and *C*_*j*_ are equal, the function value is 1; otherwise, the function value is 0.

## 5. Simulation Test and Case Analysis

The hardware environment for all experiments in this article is Intel (R) Core (TM) i5-10400F, clocked at 2.90 GHz, memory 16G, software environment Python3.7, and operating system Windows 10.

### 5.1. Simulation Test

#### 5.1.1. Data Sets

Here, we discuss 6 real networks and 2 models that simulate the real world: BA and WS, and describe the details of the network with the help of literature [34, 35]. The basic parameter structure of the network, such as the number of nodes *N*, the number of edges *M*, the average degree <*K*>, and the average shortest path length <*L*>, is shown in Table 2:

 
*Zachary Karate Network*. The Zachary Karate Network is a well-known data set [36], constructed by Zachary after two years of observing the social relations between members of a karate club in a university in the United States. There are a total of 34 nodes and 78 edges in this network. Each node represents a member. An edge between two nodes means that the two members are at least frequent friends. Due to conflicts between the manager and coach, the club is gradually split into two, and the network is naturally divided into two communities.
 
*Dolphin Social Network*. Yan et al. conducted a long-term observation on the living habits of 62 dolphins in New Zealand [37] and constructed a complex network with 62 nodes. If two dolphins often move together, then there will be an edge between the two nodes. The network has 62 nodes and 159 edges.
 
*Political Blogs Network*. Adamic and Glance analyzed 40 “A-list” blog posts in the two months before the 2004 US presidential election and studied the degree of interaction between liberals and conservatives [38, 39]. There are 1,494 nodes and 16,718 edges in the network. Each node represents a blog, and the edge represents a hyperlink between two blog pages.
 
*Power Grid Network*. The high-voltage electrical network in the western United States [40] contains 4941 nodes and 6594 edges. The transformers, substations, and generators are represented as nodes in the network, and high-voltage transmission lines are represented as connected edges.
 
*Hep-th Network*. High-energy theory collaborations' network of coauthorships between scientists posting preprints on the High-Energy Theory E-Print Archive between Jan. 1, 1995, and Dec. 31, 1999 [41, 42] contains 8631 nodes and 15751 connections.
 
*Cond-mat-2003 Network*. The astrophysics scientist cooperation network, including all preprints released between January 1, 1995, and June 30, 2005, has a total of 27,519 scientists and 116,181 edges [41].
 
*BA Network*. Barabasi and Albert proposed a scale-free network model: most nodes in the network have only a small number of edges, and a small number of nodes have high degrees, and their degree distribution obeys a power-law distribution [43]. In this paper, we set the network size *N* = 1000, *m*0 = 5, and *m* = 5.
 
*WS Network*. Watts and Strongts introduced random factors into the regular network and constructed the WS small-world network model [40]. In this paper, we set the network size *N* = 1000, *m* = 5, and *p* = 0.5.

#### 5.1.2. Node Importance Algorithm Experiment and Analysis

Based on the above 4 real networks and 2 artificial networks, in this paper, the LLSR method is compared and analyzed with the degree ranking method (Degree) which also adopts local information, the ranking method based on node degree and neighbor degree [13] (WL), H-index [14], local centrality [12] (LC), and the ranking method based on neighborhood similarity [5] (LLS). According to the sorting results of the six algorithms, nodes are removed by static attacks, and the changes in the size of the largest connected subgraph and network efficiency when the network is deliberately attacked are simulated to evaluate the accuracy of each sorting algorithm. In a static attack, the node importance index value remains the same as the calculated result value of each index in the original network and does not need to be recalculated as the network structure changes.

**Figure 2:**
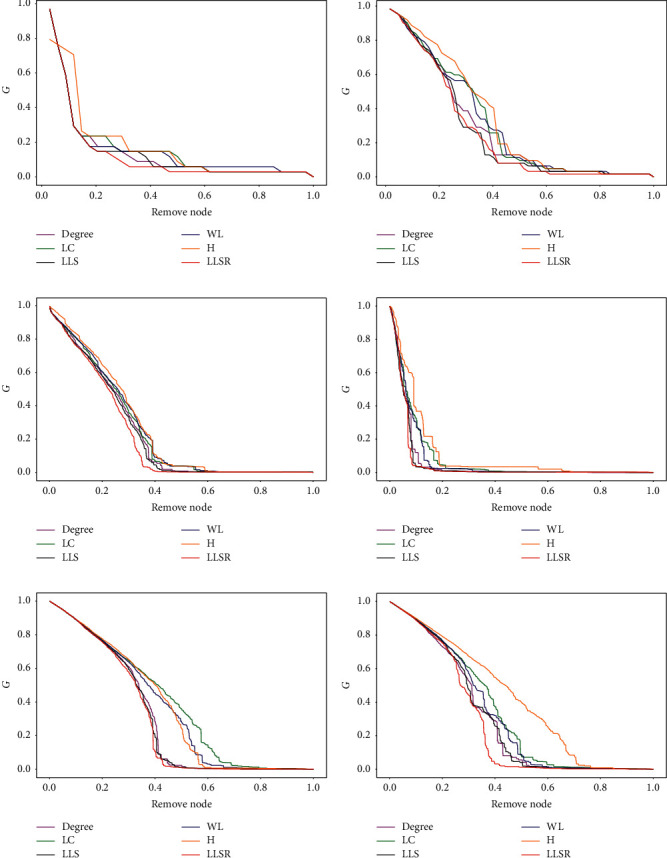
The change of the largest connected subgraph coefficient *G* after attacking important nodes of the network with different methods. (a) Karate club network. (b) Dolphin social network. (c) American political blog network. (d) American power network. (e) BA network. (f) WS network.

In the experiment of simulating the influence of deliberate attacks on the network on the largest connected subgraph of the network, the degree method (Degree), the ranking method based on node degree and neighbor degree (WL), H-index, local centrality (LC), the ranking method based on neighborhood similarity (LLS), and the LLSR method proposed in this paper are used to remove nodes in 4 real networks and 2 artificial networks. The experimental results are shown in Figures 2(a)–2(f). In Figure 2(a), except for the first removed node, among the remaining removed nodes, the nodes removed by the LLSR method are better than or equal to the best case of the other 5 methods. In Figure 2(b), when 25% to 40% of the nodes are removed from the Dolphins network, the LLSR method is slightly worse than the LLS method but better than the other four methods. After removing 40% of the nodes, the maximum connected subgraph coefficient is always in the optimal situation. In the Polbolgs network shown in Figure 2(c), the LLSR method is better than the other five methods; in the Power network shown in Figure 2(d), the LLSR method is slightly better than the LLS method, but it still performs better than the other methods. In the two networks in Figures 2(e) and 2(f), the LLSR method is significantly better than the other five methods. The LLSR method leads to the most obvious trend of decreasing the coefficient of the largest connected subgraph of the network. In Figure 2(e), the LC method appears the worst of the six methods in the BA network, which is related to the characteristics of the scale-free network. In the WS network shown in Figure 2(f), the H method performs the worst. The reason is related to the characteristics of the small-world network. The node degree distribution in the small-world network is relatively uniform. The H method has limited ability to distinguish the importance of network nodes. However, the LLSR method proposed in this paper performs the best in these two types of networks.

**Figure 3:**
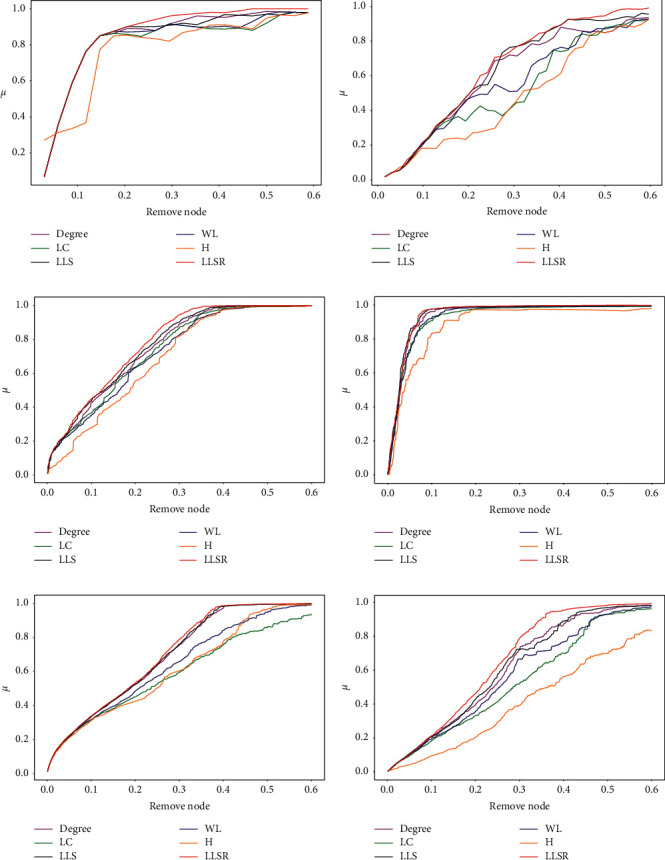
Changes in the rate of decrease in network efficiency *μ* after using different methods to attack important nodes in the network. (a) Karate club network. (b) Dolphin social network. (c) American political blog network. (d) American power network. (e) BA network. (f) WS network.

Figure 3 reflects the change in the network efficiency decline rate *μ* after using different node importance ranking algorithms to remove the network nodes. The worse the network connectivity is after the removal of important nodes, the more obvious the decline in network efficiency will be. The experimental results are shown in Figures 3(a)–3(f). In Figure 3(a), the LLSR method is worse than the other five methods when the remaining nodes are removed, except that the network efficiency of the first node is not as good as the H method. In the Dolphins network in Figure 3(b), although the LLSR method is inferior to the LLS method in some parts, it is still the best overall. In the Power network in Figure 3(d), the LLSR method is slightly inferior to the LLS method, but it is still superior to other methods. In Figures 3(c), 3(e), and 3(f), the LLSR method is at the top left of the other five methods. The LLSR method causes the largest decrease in network efficiency after removing the top nodes. The H method performed poorly in these three networks, especially the WS small-world network. From Figure 3, the LLSR method is superior to existing algorithms in most networks.

#### 5.1.3. LA-LPA Algorithm Experiment and Analysis

In the experiment, Algorithm 2 LA-LPA, LPA [44], and S-LPA [45] algorithms are run 100 times each in the above five real networks to calculate the modularity and modularity variance of the community detection results. Modularity can objectively evaluate the quality of community detection, and the fluctuation of the variance of the modularity [46] can explain the fluctuation of the community detection structure. Therefore, the modularity variance value is used to estimate the fluctuation of the community structure. The smaller the fluctuation, the better the stability of the algorithm.

*(1) Modularity Experiment Comparison Results*.  Table 3 shows the modularity comparison results on the above five real networks, and the boldface type in the table is the best result. It can be seen that the average modularity of the LA-LPA algorithm is higher than that of the S-LPA and LPA algorithms, indicating that the community quality detected by the LA-LPA algorithm has improved.

*(2) Results of Stability Comparison.* In statistics, variance can be used to calculate the difference between each variable and the overall mean [22]. In order to evaluate the stability of the detection results, this paper compares the variance of the modularity values of 100 experimental results, as shown in

(15) $$\begin{array}{c} \sigma^{2}=\frac{\sum {\left(x_{i}-X\right)}^{2}}{N}. \end{array}$$

Among them, *σ*^2^ is the population variance, *x*_*i*_ is the variable, *X* is the population mean, and *N* is the total number of samples. The lower the variance, the better the stability of the surface results.

Table 4 shows the experimental results of the algorithm stability of the Algorithm 2 LA-LPA, LPA, and S-LPA algorithms on the real network. The boldface in the table is the best result. It can be concluded that the LA-LPA algorithm of this paper can obtain more stable results in most networks; that is, the LA-LPA algorithm is better than other algorithms in the stability of mining potential teams.

### 5.2. Crowdsourcing Designer Network Example Analysis

An example analysis of the algorithm proposed in this paper is carried out in a network of crowdsourcing designers. The crowdsourcing designer network uses the crowdsourcing designer WeChat group as the analysis object. By using the conversation communication data within the WeChat group as the analysis text, the data is preprocessed into a network structure. There are a total of *N* = 82 designers in the network, *M* = 203 edge relations, <*K*> = 4.95, and <*L*> = 3.07.

#### 5.2.1. Core Personnel Identification

**Figure 4:**
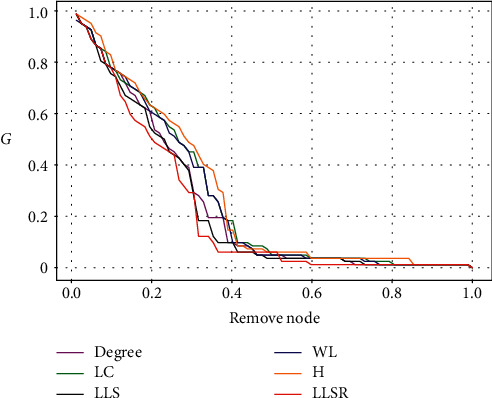
The change of the largest connected subgraph coefficient *G* after attacking important nodes of the crowdsourcing designer network with different methods.

**Figure 5:**
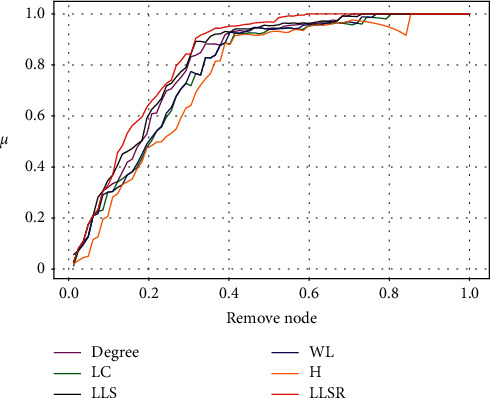
Changes in the rate of decrease in network efficiency *μ* after using different methods to attack important nodes in the crowdsourcing designer network.

Discovering the core personnel in the network can strengthen the robustness of the network through targeted protection of these core personnel; in turn, it will also cause the entire network to collapse by deliberately attacking these core personnel. In addition, choosing core personnel can also make information spread faster on the network. The LLSR algorithm mentioned in this article and the other five algorithms mentioned above are used to identify the core personnel. The change of the maximum connected subgraph coefficient *G* of the network and the change of the network efficiency drop rate are shown in Figures 4 and 5. It can be seen that after the LLSR algorithm deliberately attacked some key nodes, the maximum connected subgraph and network efficiency of the entire network were in the lowest state. After attacking 60% of the nodes, the entire network is in a state of paralysis, the network efficiency is 0, and the maximum connected subgraph coefficient is almost 0. This verifies the effectiveness of the LLSR algorithm. Compared with other algorithms, LLSR can better distinguish the core personnel in the network.

#### 5.2.2. Potential Team Detection

The LA-LPA, LPA, and S-LPA algorithms are applied to the constructed crowdsourced designer network, and the results after 100 runs are shown in Table 5. It can be seen that the modularity obtained by the LA-LPA algorithm is large, which is 9.4% higher than that of the LPA algorithm, and the modularity variance is 0, indicating that the team detected by the algorithm is of high quality, compact structure, and algorithm stability.

Respectively count the teams detected according to the algorithm in this paper and count the number of cooperating personnel, average degree, and team density, and calculate the average value. The results are shown in Table 6. The size of each team varies, and each team contains an average of 20.5 designers. The average degree within each team is 3.41, indicating that a designer communicates with 3.41 other designers on average. The greater the network density, the closer the relationship between the network nodes, the overall density of the network is 0.06, and the average team density is 0.24, indicating that the relationship between team members is close.

The analysis of the structure of the crowdsourcing designer network found that there are close connections between certain nodes in the network, which makes these nodes combine into small teams. From the test results, it is found that some cooperation teams have a large number of people, and some have a small number of teams, because, in the network, the team does not have a unified size and clear boundary. In addition, further analysis of the communication between the teams can be seen in Table 7 where the distribution of the teams is obvious, not in isolation, but in cross-team communication with each other. In the entire network, there are nodes between teams that act as bridges, connecting these teams into a large network of designers. The internal cooperation of the team is close, and the cooperation between the teams is relatively sparse, which verifies the rationality of the algorithm detection in this paper.

## 6. Conclusion

In the designer network, the core designer is in an advantageous position in the network. In order to distinguish the designer's importance in the network, not only the network topology but also the trust degree of information propagation on the network is considered, because people's opinions and behaviors are often affected by neighbors, so this paper proposes a node importance ranking method LLSR that regards nodes as the output of the neural network and neighboring nodes' neighborhood similarity as the input of the neural network. In the case of node local network topology overlap, through information propagation, the similarity of nodes is revised. This method only needs to calculate the neighbor information within the two hops of the node to calculate its importance, which is of practical significance for large-scale networks to find key nodes. Exploring the potential teams and their structures in the network is of great research significance for studying the rules of information dissemination and providing accurate recommendations. For this reason, this paper proposes a LA-LPA algorithm based on node importance and node similarity, which replaces the random node update sequence with the node importance sequence, and avoids the randomness of label propagation by selecting the node label with the highest similarity.

In the experiment of a crowdsourcing designer network constructed by a virtual community and 6 other real networks and 2 artificial networks, network efficiency reduction rate and largest connected subgraph coefficients are used to verify the LLSR algorithm proposed in this paper, and modularity and variance are used as evaluation criteria to verify the LA-LPA algorithm proposed in this paper. Experimental results show that the LLSR algorithm in this paper is superior to the degree method based on local information, the WL ranking method based on node degree and its neighbor degree, the H-index, the local centrality LC method, and the LLS ranking method based on neighborhood similarity. The LA-LPA algorithm is superior to the LPA and S-LPA algorithms in terms of modularity and stability.

For the evaluation of the importance of nodes, the ultimate goal is to sort the nodes according to the importance of the evaluation criteria and prepare for the follow-up research, such as constructing an infectious disease model to understand and predict the mode in which information is spread in the network [47, 48]. In a virus transmission network, if some important nodes in the network can be isolated in time, the virus transmission can be effectively suppressed [49, 50]. For the mining of potential communities and their structures, collaborative tasks can be provided to communities to lay the foundation for building a big data mining framework to provide more effective resource recommendation services in the future.

Although the two methods proposed in this paper can identify the core designers of the designer network and the potential team structure in the network, this paper only analyzes the core designers in a single-layer network, and the actual network may be related to each other. Once a node is destroyed, a butterfly effect will occur and the entire network will be paralyzed. And designers not only belong to a team but also may belong to multiple teams, so how to find core designers in the interconnection network analysis and how to mine the overlapping teams is our next research direction.

## Figures and Tables

**Algorithm 1:**
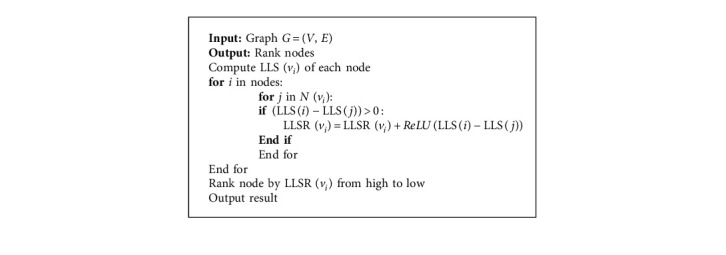
*LLSR* algorithm.

**Algorithm 2:**
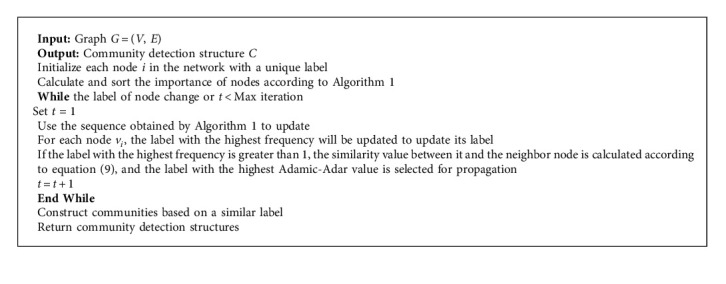
LA-LPA.

**Table 1:** Time complexity comparison.

Evaluation method name	Locality	Time complexity
LLSR	Local	*O*(*n*(*k*^2^+*k*))
LLS	Local	*O*(*nk*^2^)
Degree	Local	*O*(*n*)
WL	Local	*O*(*m*+*nk*)
BC	Global	*O*(*n*^3^)
CC	Global	*O*(*n*^3^)

**Table 2:** Topological characteristics of 6 real networks and 2 artificial networks.

Data sets	*N*	*M*	<*K*>	<*L*>
Karate	34	78	4.59	2.41
Dolphins	62	259	5.13	3.36
Polbolgs	1490	16718	22.44	2.74
Power	4941	6594	2.67	18.99
Hep-th	8361	15751	3.77	3.42
Cond-mat-2003	27519	116181	8.44	5.77
BA	1000	4975	9.95	2.99
WS	1000	2000	4.00	5.59

**Table 3:** Modularity comparison of 6 real network experiment results.

Data sets		Karate	Dolphins	Polblogs	Power	Hep-th	Cond-mat-2003
Average value	LPA	0.3570	0.4883	0.4007	0.5948	0.6786	0.6080
	S-LPA	0.3547	0.4986	0.0013	0.6278	0.6879	0.6361
	LA-LPA	**0.3764**	**0.5091**	**0.4260**	**0.7316**	**0.7454**	**0.6878**

**Table 4:** Comparison of the stability of LA-LPA, LPA, and S-LPA algorithms.

Data sets		Karate	Dolphins	Polblogs	Power	Hep-th	Cond-mat-2003
Variable	LPA	0.0054	0.0012	0.0102	1.59 × 10^−5^	6.37 × 10^−5^	3.54 × 10^−5^
	S-LPA	1.23 × 10^−32^	1.23 × 10^−32^	4.70 × 10^−38^	1.23 × 10^−32^	1.97 × 10^−31^	4.93 × 10^−32^
	LA-LPA	1.23 × 10^−32^	**0**	1.23 × 10^−32^	1.23 × 10^−32^	1.23 × 10^−32^	1.11 × 10^−31^

**Table 5:** Results of different algorithms in crowdsourcing designer networks.

Evaluation indicator	LPA	S-LPA	LA-LPA
Average number of communities	8.96	9	4
*Q*	0.3692	0.3503	0.4038
Variable	0.0068	3.08 × 10^−33^	0

**Table 6:** Crowdsourcing designer network team information.

Team number	The number of people in the team	Number of internal team relationships	Average degree within the team	Team density
1	7	11	3.14	0.52
2	20	30	3	0.16
3	24	46	3.83	0.17
4	31	57	3.68	0.12
Average	20.5	36	3.41	0.24

**Table 7:** Exchange information between teams.

Team number	1	2	3	4
1	11	4	5	5
2	4	30	7	25
3	5	7	46	13
4	5	25	13	57

## Data Availability

The data used to support the findings of this study are available from the corresponding author upon request.
